# Maintenance therapy improves the survival outcomes of patients with metastatic nasopharyngeal carcinoma responding to first-line chemotherapy: a multicentre, randomized controlled clinical study

**DOI:** 10.1007/s00432-022-04341-2

**Published:** 2022-09-08

**Authors:** Ying Lu, Haixin Huang, Hui Yang, Xiaohua Hu, Meilian Liu, Changjie Huang, Xianbin Feng, Xishan Chen, Zhou Jiang

**Affiliations:** 1 Department of Oncology, The Fourth Affiliated Hospital of Guangxi Medical University, No.1 Liushi Road, Liuzhou, 545000 Guangxi China; 2 Department of Oncology, The First Affiliated Hospital of Guangxi Medical University, Nanning, 530000 China; 3 Department of Oncology, Affiliated Hospital of Guilin Medical College, Guilin, 541000 China; 4 Department of Oncology, The Third Affiliated Hospital of Guangxi Medical University, Nanning, 530000 China; 5 Department of Oncology, Liuzhou Hospital of Traditional Chinese Medicine, Liuzhou, 545000 China

**Keywords:** Nasopharyngeal carcinoma, Tegafur/gimeracil/oteracil, Maintenance therapy, Epstein‒Barr virus-DNA, Human serum amyloid A

## Abstract

**Purpose:**

To explore the safety and role of tegafur/gimeracil/oteracil (S1) maintenance therapy (MT) in metastatic nasopharyngeal carcinoma (NPC) patients after response to first-line chemotherapy and to assess outcome-associated biomarkers.

**Methods:**

This was a multicentre, open-label, randomized controlled study involving metastatic NPC patients recruited (from May 2015 to May 2019) at five hospitals in China. The participants were randomized to S1-MT (receiving S1 MT until disease progression or intolerance) or non-MT (followed up until disease progression) groups. The primary endpoint was the progression-free survival (PFS). The secondary endpoints were the overall survival (OS), the correlation between EBV-DNA, serum amyloid A (SAA) status, and outcomes after the first-line chemotherapy, and safety.

**Results:**

The median follow-up was 24.3 months; 88 and 95 participants were evaluable in the S1-MT and non-MT groups, respectively. Compared with non-MT, S1-MT prolonged PFS (16.9 vs. 9.3 months, *P* < 0.001) and OS (33.6 vs. 20.6 months, *P* < 0.001). Regardless of their EBV-DNA status after first-line chemotherapy, participants were able to benefit from S1 MT, but EBV-DNA-positive participants benefited more significantly (PFS: HR = 0.600, 95% CI = 0.373–0.965, *P* = 0.035; OS: HR = 0.393, 95% CI = 0.227–0.681, *P* = 0.001). MT only improved PFS and OS in patients with an SAA decline after first-line chemotherapy (PFS: HR = 0.570, 95% CI = 0.350–0.919, *P* = 0.021; OS: HR = 0.404, 95% CI = 0.230–0.709, *P* = 0.002). The median S1 treatment was 23 cycles. Grade 1–2 skin pigmentation, oral mucositis, and hand and foot syndrome were the main adverse reactions.

**Conclusion:**

For metastatic NPC patients with first-line chemotherapy response, S1 MT can improve PFS and OS, with good tolerability. EBV-DNA and SAA can better help us identify patients who can benefit from MT after standard treatment.

**Trial registration:**

The study protocol was registered at the Chinese Clinical Trial Registry (ChiCTR-IOR-16007939).

**Supplementary Information:**

The online version contains supplementary material available at 10.1007/s00432-022-04341-2.

## Introduction

Nasopharyngeal carcinoma (NPC) arises from the epithelium of the nasopharynx (Kamran et al. 2015). Epstein‒Barr virus (EBV) infection is associated with NPC, particularly the undifferentiated nonkeratinizing subtype in endemic regions (Chua et al. 2016; Tsao et al. 2014). Despite the excellent local control of NPC owing to advances in radiotherapy technology, 20–30% of NPC patients will progress to distant metastasis (Chan et al. 2012; Lee et al. 2015*; NCCN Clinical Practice Guidelines in oncology (NCCN Guidelines). Head and Neck Cancers. Version 3.2021*, 2021). Presently, platinum-based combined chemotherapy is the main first-line treatment option. The first-line treatment option with gemcitabine plus cisplatin (GP) achieved better PFS (7.0 vs. 5.6 months, *P* < 0.0001) and OS (29.1 vs. 20.9 months, *P* < 0.0025) than a classic regimen with fluorouracil plus cisplatin (PF) in patients with recurrent or metastatic NPC (Zhang et al. 2016). Nevertheless, the overall outcome of first-line chemotherapy for metastatic NPC remains disappointing, and the median PFS is only 2.7–7.2 months even after combination with EGFR monoclonal antibodies (Chan et al. 2005; Chua et al. 2016; Jin et al. 2012). Thus, better treatment strategies are still needed to delay disease progression in NPC patients achieving disease control (DC) from first-line chemotherapy.

Maintenance therapy (MT) is a treatment strategy that continues drug treatment when the tumour burden reaches the minimum to delay progression and prolong OS; this approach has been successful in treating metastatic lung, breast, and colorectal cancers (Gerber and Schiller 2013; Loree et al. 2018; Luo et al. 2016; Surmeli et al. 2015). The chronic administration of low-dose chemotherapy not only directly affects tumour cells, but also acts on the cell microenvironment, by inhibiting tumour angiogenesis, or promoting the immune response (Cazzaniga et al. 2021). The oral 5-FU derivatives have been used for auxiliary intensive treatment (Ke et al. 2014; Suzuki et al. 2016; Wang et al. 2021) or extension of the treatment of metastatic diseases (Haag et al. 2017; Luo et al. 2016; Surmeli et al. 2015). 5-FU is also a traditional classic drug for NPC, and its derivative drugs achieve good effects. After standard treatment comprising concurrent chemoradiotherapy with or without induction chemotherapy, capecitabine maintenance increased the 3-year disease-free survival (DFS) in high-risk locally advanced NPC patients by 9.6% and the OS by 4.7% with good tolerability (Chen et al. 2021). In newly diagnosed metastatic NPC and metastatic cases after definitive chemoradiotherapy, MT with 5-FU derivatives also achieved good survival benefits, and the 3-year PFS and OS rates were 47.6% and 87.7%, respectively (Guo et al. 2020). Nevertheless, the role of MT in progressive metastatic NPC remains unclear.

S1 is a third-generation fluorouracil derivative oral anticancer agent with excellent oral bioavailability and a similar effect to 5-FU continuous intravenous infusion (Chhetri et al. 2016; Yokota et al. 2011). S1 has been shown to have good clinical efficacy and tolerability in metastatic NPC (Peng et al. 2014, 2016, 2017). For second-line S1 monotherapy in patients with recurrent/metastatic NPC, the median time to progression (TTP) was 5.6 months and the incidence of grade 3 adverse reactions was only 7.7% (Peng et al. 2014). Thus, S1 MT in NPC has a clinical basis and warrants further research.

Biomarkers could be useful in predicting who might benefit from further treatments. Changes in the peripheral blood EBV-DNA load are related to changes in NPC burden, which might be more sensitive than imaging examinations in suggesting disease changes (Shen et al. 2015; You et al. 2019). A high EBV-DNA load after treatment is associated with adverse NPC outcomes, and the residual EBV-DNA load might be a useful indicator of the rationality of subsequent adjuvant chemotherapy (Yip et al. 2014). Serum amyloid A (SAA) comprises a group of proteins encoded by multiple genes and is associated with tumour burden and prognosis (Lin et al. 2019; Moshkovskii, 2012). SAA rises rapidly when NPC progresses and can be used as a potential disease monitoring marker (Chen et al. 2018; Cho et al. 2004). The 5-year PFS of NPC patients with high SAA levels was significantly lower than that of patients with low SAA (≤ 4.28 mg/L) (64.5% vs. 73.1%, *P* = 0.013) (Luo et al. 2016). The integrated classification of SAA and EBV-DNA can better predict the OS prognosis of NPC (Li et al. 2020).

The present prospective, multicentre, randomized controlled study was performed in metastatic NPC patients benefiting from the first-line chemotherapy to explore clinical value of S1 MT and the correlation between EBV-DNA, SAA status, and the benefits of MT. The results could help screen the patients who could achieve potential benefits from MT.

## Materials and methods

### Study design and participants

This was a multicentre, open-label, randomized controlled study involving metastatic NPC patients recruited from May 2015 to May 2019 at the Cancer Treatment Center of the Fourth Affiliated Hospital of Guangxi Medical University, Chemotherapy Department of the First Affiliated Hospital of Guangxi Medical University, Oncology Department of the Affiliated Hospital of Guilin Medical University, Oncology Department of the Third Affiliated Hospital of Guangxi Medical University, and Oncology Department of the Liuzhou Traditional Chinese Medicine Hospital. The study was reviewed and approved by the Institutional Review Board of the Fourth Affiliated Hospital of Guangxi Medical University (PJK2015221). The clinical trial registration number is ChiCTR-IOR-16007939. Written informed consent was obtained from all participants prior to any study-related procedures. The study was conducted according to the tenets of the Declaration of Helsinki and Good Clinical Practice.

The inclusion criteria were (1) pathologically diagnosed nasopharyngeal undifferentiated nonkeratinizing carcinoma, (2) prior potentially curative treatment, (3) clinically/pathologically diagnosed distant metastasis, with at least one measurable lesion, (4) first-line chemotherapy with ≥ 4 cycles, and efficacy evaluation of clinical benefit based on the DC, (5) Eastern Cooperative Oncology Group (ECOG) score < 2 points, and (6) giving written consent to participate in the study. The exclusion criteria were (1) other malignant tumours, (2) chemotherapy contraindications, (3) allergies to any of the drugs in the regimen, or (4) inability to take oral drugs or attend the follow-up visits.

### Randomization and blinding

All eligible participants were randomly assigned in a 1:1 ratio to the MT and non-MT groups using a central Internet-based randomization system prepared using a random table number and maintained by a third-party biostatistician. The researchers and participants were not blinded in the present study.

### Intervention

In the MT (S1-MT) group, the participants received S1 (Jiangsu Hengrui Pharmaceuticals Co., Ltd., China) MT (body surface area < 1.5 m^2^, 50 mg/time; body surface area > 1.5 m^2^, 60 mg/time; bid; days 1–14 of each 21-days cycle) until disease progression or intolerance. The participants in the non-MT group were followed up until disease progression. In the S1-MT group, according to the NCI-CTCAE 3.0 toxicity classification criteria (Trotti et al. 2003), if there were two consecutive adverse reactions of grade ≥ 3 that could not be improved by treatment or discontinuation (≤ 2 weeks), then S1 was reduced to the dose level of the next surface area. If the dose had to be reduced > 2 times, treatment was terminated. In the first S1 medication, no routine antiemetic or granulocyte colony-stimulating factor was administered for preventive treatment. The subsequent cycles were adjusted according to individual conditions.

### Outcomes

The primary endpoint was PFS. The secondary endpoints were OS, the correlation between EBV-DNA, SAA status, and outcomes after the first-line chemotherapy, and the adverse reactions to S1 MT. PFS was the time from the end of the first-line chemotherapy to the first instance of disease progression. OS was defined as the time from the end of the first-line chemotherapy to death from any cause.

The efficacy evaluation was performed according to the response evaluation criteria in solid tumours (RECIST 1.1) (Schwartz et al. 2016). Adverse reactions were evaluated according to the NCI-CTCAE 3.0 classification criteria for common adverse drug reactions (Trotti et al. 2003).

### Detection of EBV-DNA and SAA

EBV-DNA and SAA detection was performed by designated personnel with the same source reagents and the same detection standards. Quality control checks between laboratories were carried out regularly. Peripheral blood samples before, during, and after the first-line chemotherapy and at each follow-up point (once every 3 months) during the MT/follow-up period were collected.

The PCR-fluorescent probe method was used to determine the plasma EBV-DNA levels. EBV-DNA detection was performed with 2 ml of venous blood within 1 h. The lower limit of detection was < 5.0 × 10^2^ IU/ml. The reagents were from Daan Gene Co., Ltd. (China) of Sun Yat-Sen University. EBV-DNA was negative if it was below the lower limit of detection and had no amplification; otherwise, it was positive. The double antibody sandwich method was used to determine the SAA levels. The absorbance (OD value) was measured with a microplate reader at a wavelength of 450 mm. The SAA levels were calculated from the standard curve. The reagents were provided by Shanghai Yanjin Biotechnology Co., Ltd.

### Follow-up

The same evaluation protocol was used. Blood tests, such as routine blood and liver function tests, were performed before and after each cycle of S1-MT, and were simultaneously performed at each imaging evaluation for non-MT patients. Computed tomography (CT) and/or magnetic resonance imaging (MRI) were performed every 3 months for efficacy evaluation. CT was used to evaluate the lung and abdomen. MRI was used to evaluate the nasopharynx and bone, and emission computed tomography (ECT) was used to identify bone metastases if necessary.

### Statistical analysis

The statistical analysis scheme was based on clinical superiority. It was expected that the primary endpoint PFS would be increased by > 10% in the S1-MT group. Using *α* = 0.05, *β* = 0.20, and 10% drop-off rate, the sample size was determined to be 102 participants in each group, for a total sample size of 204. SPSS 22.0 (IBM, Armonk, NY, USA) was used for statistical analysis. Continuous data were tested for normal distribution using the Shapiro‒Wilk test. Nonnormally distributed continuous data are presented as the median (range) and were analysed using the Mann‒Whitney *U* test. Categorical data are presented as *n* (%) and were analysed using the Chi-square test. The Kaplan‒Meier survival curve and log-rank analysis were used for survival analysis. The Cox risk model was used for univariable and multivariable analyses of survival and prognosis. Two-sided *P* values < 0.05 were considered statistically significant.

## Results

### Baseline characteristics of the participants

**Fig. 1:**
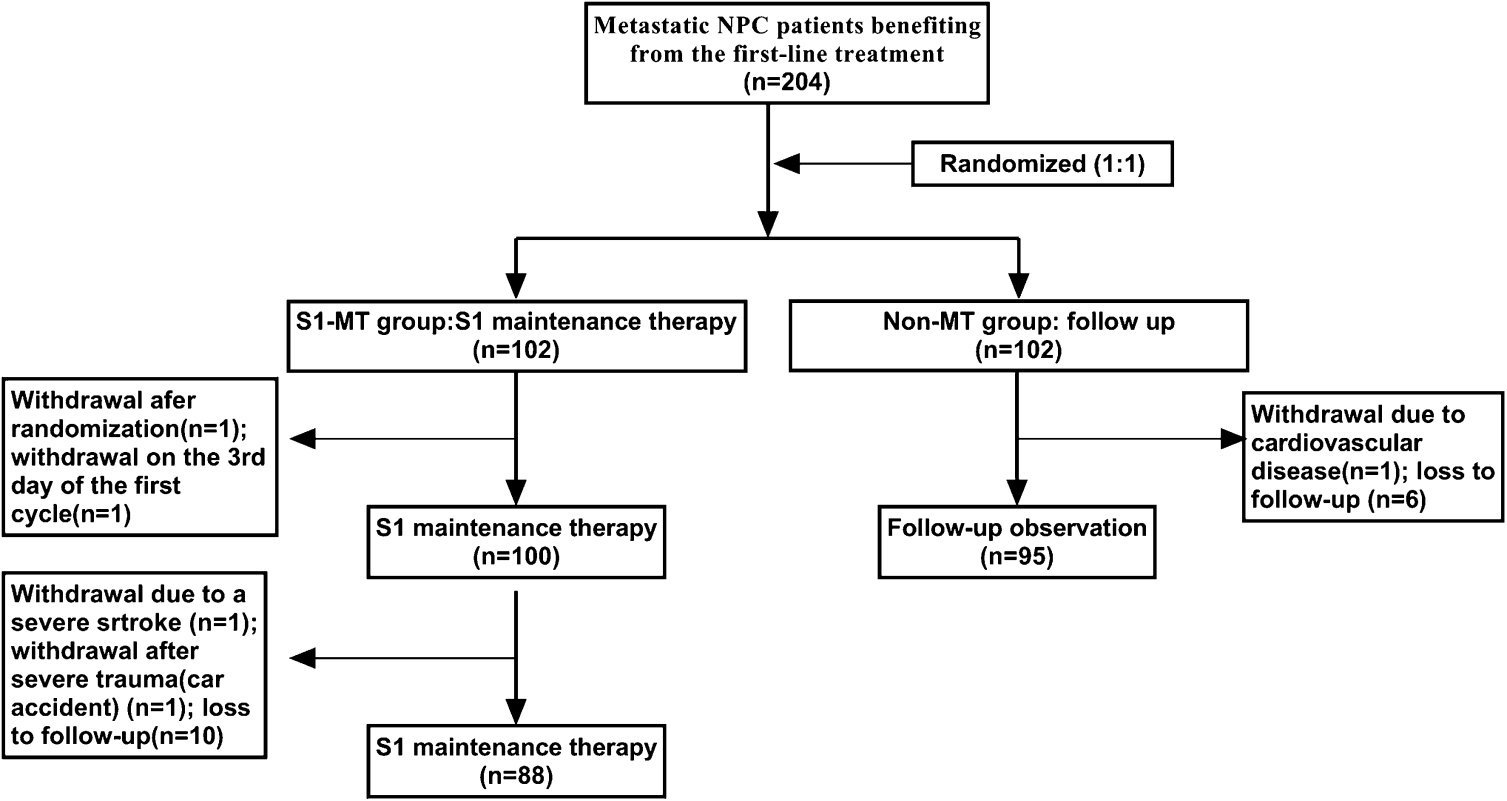
Flowchart of the patients

A total of 204 participants were enrolled, with 183 being evaluable, including 88 in the S1-MT group and 95 in the non-MT group (Fig. 1). The median age was 49 years (22–68 years). There were 132 males, and 51 females, with a ratio of 2.6:1. All patients were treated by IMRT in the therapy of initial diagnosis. The first-line chemotherapy was platinum-based combination therapy. There were no statistically significant differences in any of the baseline characteristics between the two groups (all *P* > 0.05), as shown in Table 1.

**Table 1:** Comparison of clinical characteristics between two groups

Characteristics	S1-MT (*n* = 88)	Non-MT (*n* = 95)	*P*
Age, years, median (range)	46 (22–65)	48 (29–68)	0.314
Sex			0.135
Male	68 (77.3%)	64 (67.4%)	
Female	20 (22.7%)	31 (32.6%)	
Clinical stage of initial diagnosis (AJCC 7th)			0.439
II	3 (3.4%)	6 (6.3%)	
III	40 (45.5%)	48 (50.5%)	
IVa/b	45 (51.1%)	41 (43.2%)	
The treatment of initial diagnosis			0.179
IC + CCRT	70 (79.6%)	65 (65.4%)	
CCRT	10 (11.4%)	21 (22.1%)	
CCRT + ACT	7 (8.0%)	6 (6.3%)	
RT alone	1 (1.1%)	3 (3.2%)	
Tumour metastasis site			0.738
Single organ	15 (17.1%)	18 (19.0%)	
Multiple organs	73 (82.9%)	77 (81.0%)	
Liver metastasis			0.303
Yes	42(45.7%)	50(54.3%)	
No	46(50.5%)	45(49.5)	
First-line chemotherapy after metastasis			0.258
GEM + Platinum	45 (51.1%)	60 (63.2%)	
PTX/DOC + Platinum	34 (38.6%)	28 (29.5%)	
5-FU + Platinum	9 (10.2%)	7 (7.4%)	
ECOG of enrolling			0.393
0	8 (9.1%)	5 (5.3%)	
1	80 (90.9%)	90 (94.7%)	
EBV-DNA of enrolling			0.522
Positive	46 (52.3%)	49 (51.6%)	
Negative	42 (47.7%)	46 (48.4%)	
SAA of enrolling			0.544
Continuous decline	41 (56.6%)	44 (51.6%)	
Stable	47 (53.4%)	51 (53.7%)	

*MT* maintenance therapy, *AJCC* American Joint Cancer Committee, *IC* induction chemotherapy, *CCRT* concurrent chemoradiation, *ACT* adjuvant chemotherapy, *RT* radiotherapy

### Analysis of efficacy

**Fig. 2:**
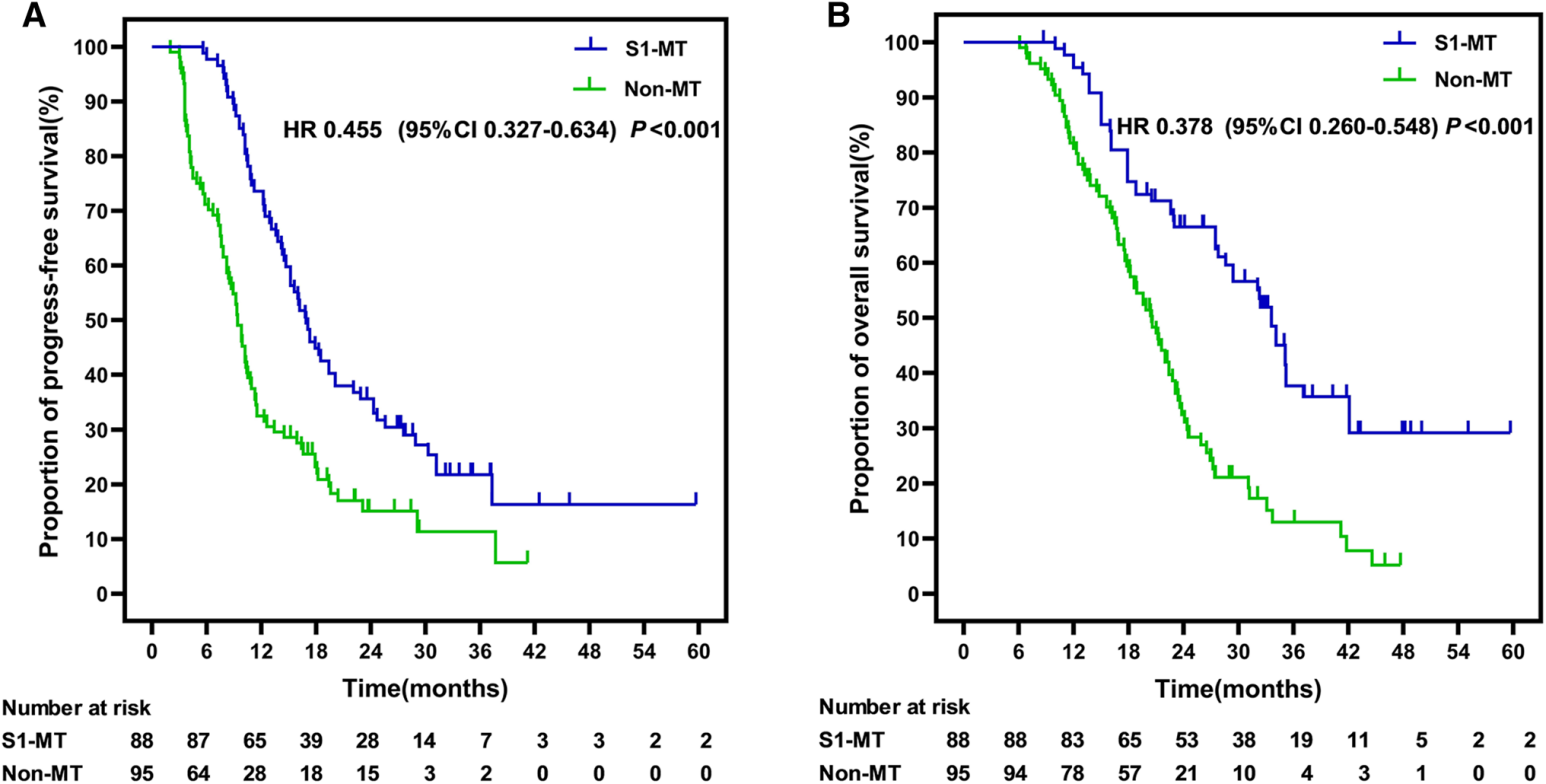
Comparison of PFS (**A**) and OS (**B**) Kaplan–Meier survival curves between the two groups

Follow-up was censored on May 31, 2021, with a median of 24.3 (6.1–59.8 months). The 2-year PFS and OS were higher in the S1-MT group (PFS 25.0% and OS 43.2%) than in the non-MT group (PFS 14.7% and OS 20.0%). Compared with the non-MT group, S1-MT significantly prolonged median PFS (16.9 vs. 9.3 months, *P* < 0.001) and median OS (33.6 vs. 20.6 months, *P* < 0.001). The OS benefit was more significant after 24 months (Fig. 2). S1 MT significantly lowered the risk of adverse outcomes related to PFS and OS (PFS: HR = 0.445, 95% CI 0.327–0.634, *P* < 0.001; OS: HR = 0.378, 95% CI 0.260–0.548, *P* < 0.001).

**Fig. 3:**
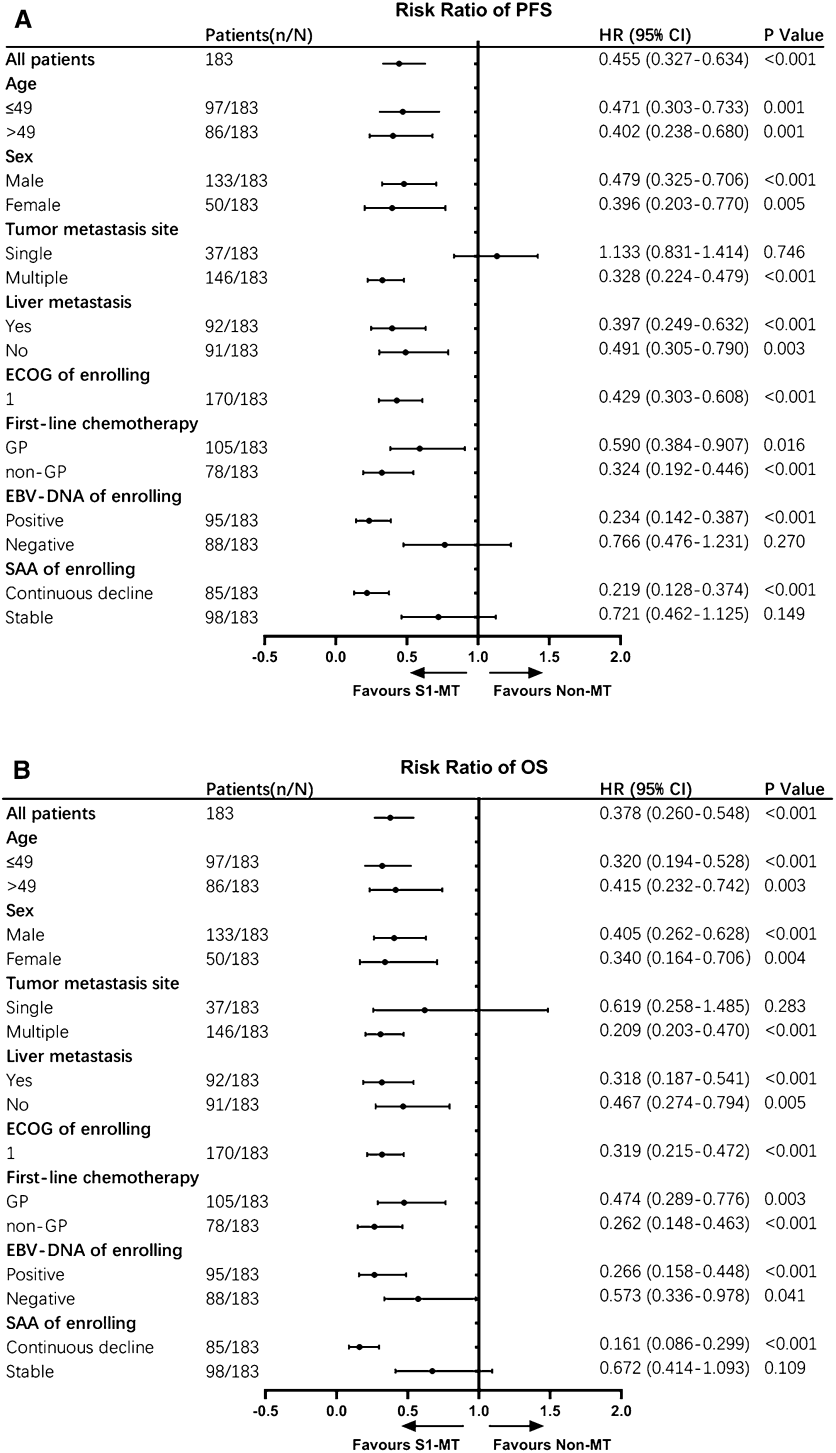
Subgroup analysis of PFS (**A**) and OS (**B**) in the intention-to-treat population

Across the patient subgroups, we observed a consistent benefit in favour of S1-MT in terms of PFS and OS, including for different ages, sexes, liver metastases, ECOG scores, first-line treatment regimens, and multisite metastases (all *P* < 0.05) (Fig. 3). However, the patients with single-site metastases or stable SAA did not benefit from maintenance therapy.

### Analysis of correlation between the EBV-DNA, SAA, and outcomes

**Fig. 4:**
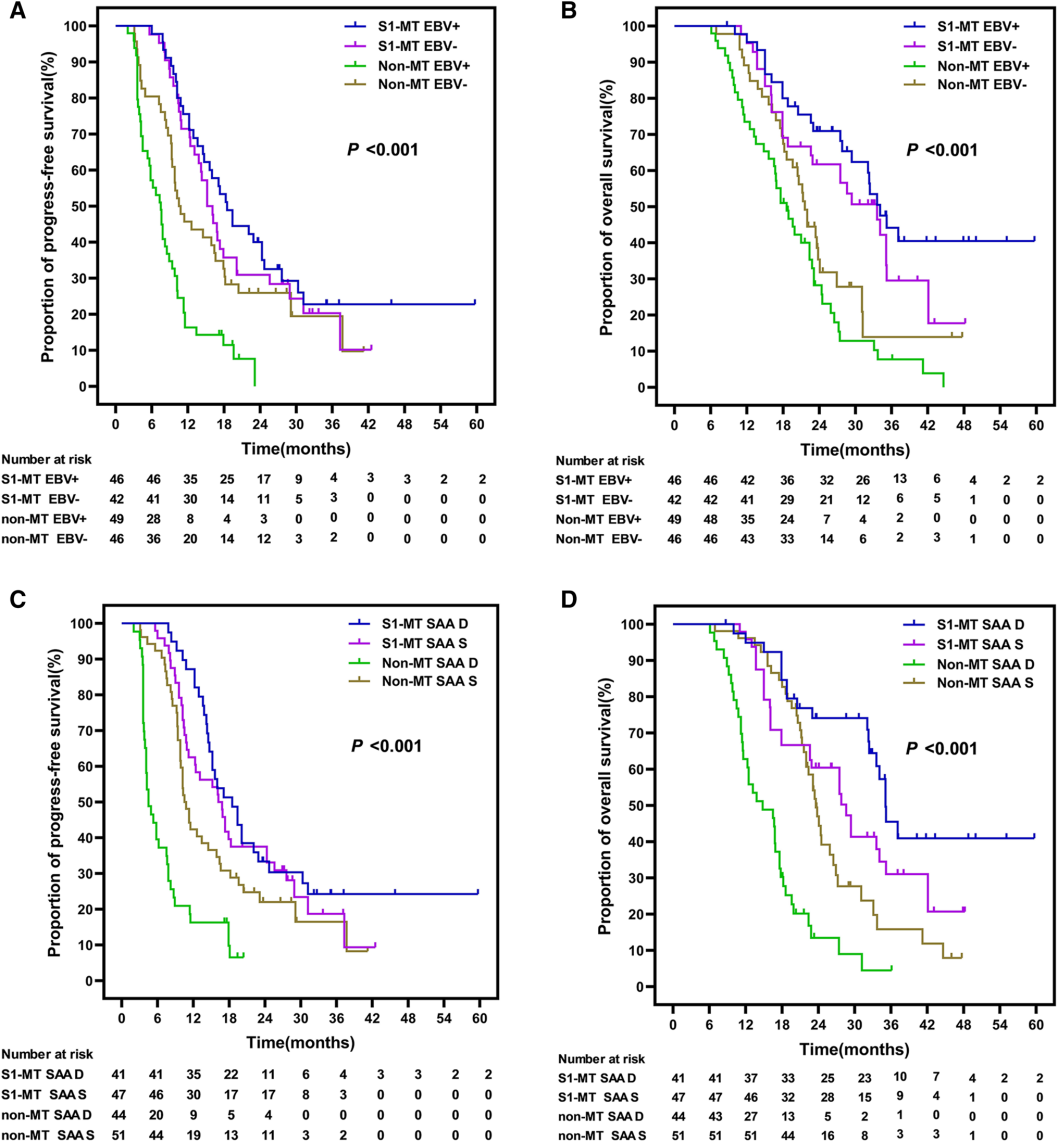
Comparison of progression-free survival (PFS) and overall survival (OS) Kaplan–Meier survival curves of patients with different EBV-DNA status (**A**/**B**) and SAA status (**C**/**D**) between the two groups

EBV-DNA was detected after first-line chemotherapy in 51.9% (95/183) of all participants. The participants who received S1 MT had better PFS and OS. Indeed, the median PFS and OS in the EBV-DNA-positive and EBV-negative participants in the S1-MT group and the EBV-DNA-positive and EBV-negative participants in the non-MT group were 18.5, 15.2, 7.5, and 10.5 months (*P* < 0.001) and 34.1, 33.6, 18.6, and 23.6 months (*P* < 0.001), respectively (Fig. 4A, B). Compared with EBV-DNA-negative non-MT participants, EBV-DNA-positive non-MT participants had a significantly higher adverse risk of PFS (HR = 2.450, 95% CI 1.565–3.836, *P* < 0.001), but EBV-DNA-positive MT participants had lowered the adverse risk of PFS by 40.0% (HR = 0.600, 95% CI 0.373–0.965, *P* = 0.035) and the adverse risk of OS by 60.7% (HR = 0.393, 95% CI 0.227–0.681, *P* = 0.001) lower adverse risk of PFS (Table S1). This means that detectable EBV-DNA following first-line chemotherapy was a prognostic adverse factor for PFS and OS, but that MT with S1 would improve the PFS and OS in such patients.

In total, 46.4% (85/183) of patients during first-line treatment had a continuous decrease in SAA levels. The median PFS and OS of the participants with continuously decreased vs. stable SAA in the S1-MT group, and those with continuously decreased vs. stable SAA in the non-MT group were 17.1 vs. 15.2 and 4.5 vs. 10.2 months (*P* < 0.001) and 34.1 vs. 27.5 and 13.2 vs. 21.6 months (*P* < 0.001), respectively. S1 MT significantly improved the PFS and OS of participants with continuously decreased SAA during first-line treatment (Fig. 4C, D). Compared with non-MT participants with stable SAA after first-line treatment, S1 MT did not significantly improve the risk of adverse outcomes in participants with stable SAA, but the adverse risks of PFS and OS in participants with continuously decreased SAA were significantly reduced (PFS: HR = 0.570, 95% CI 0.350–0.919, *P* = 0.021; OS: HR = 0.404, 95% CI 0.230–0.709, *P* = 0.002) (Table S1).

### Univariate analysis and multivariate analysis for PFS and OS of metastatic NPC patients benefiting from the first-line treatment

Univariate analysis of the Cox risk ratio model confirmed that MT, liver metastasis, EBV-DNA, and SAA were prognostic factors for PFS and OS in metastatic NPC patients benefiting from first-line treatments (all *P* < 0.05) (Table S2). However, the multivariable analysis showed that S1-MT (PFS: HR = 0.326, 95% CI 0.255–0.473, *P* < 0.001; OS: HR = 0.375, 95% CI 0.239–0.590, *P* < 0.001) and liver metastasis (PFS: HR = 0.679, 95% CI 0.484–0.952, *P* = 0.025; OS: HR = 0.473, 95% CI 0.310–0.722, *P* = 0.001) were associated with the PFS and OS outcomes in the participants, and the SAA status only showed correlations with OS outcomes (HR = 0.621, 95% CI 0.392–0.984, *P* = 0.042) (Table S2).

### MT-associated adverse reactions

In the S1-MT group, the median S1 treatment was 23 cycles (8–71 cycles). The main adverse reactions were skin pigmentation, oral mucositis, and hand and foot syndrome of grade 1–2. Grade 4 toxic reactions did not occur (Table 2). Adverse reactions mainly occurred within 9–10 days of medication and were alleviated during the 7-day pause in each cycle. Some participants could tolerate the adverse effects after dose adjustments or short-term treatment delays (< 2 weeks). No participants were excluded due to adverse reactions. During treatment, 12 (13.6%) participants had two consecutive dose adjustments. Twenty-one (23.9%) participants had more than two short-term medication delays.

**Table 2:** Adverse reaction of S1 maintenance therapy

Adverse reaction	Grade of adverse reaction (n = 88)			
	0	1	2	3
Pigmentation of skin	2 (2.3%)	86 (97.7%)	0	0
Hand and foot syndrome	4 (4.5%)	69 (78.4%)	12 (13.6%)	3 (3.5%)
Oral mucositis	33 (37.6%)	52 (59.1%)	3 (2.3%)	0
Nausea	51 (58.0%)	35 (39.8%)	2 (2.2%)	0
Vomiting	72 (81.8%)	16 (18.2%)	0	0
Neutropenia	32 (26.3%)	46 (52.3%)	7 (8.0%)	3 (3.4%)
Anemia	37 (42.1%)	48 (54.5%)	3 (3.4%)	0
Thrombocytopenia	75 (85.2%)	12 (13.6%)	1 (1.2%)	0
Liver function damage	79 (89.8%)	8 (9.0%)	1 (1.2%)	0

## Discussion

The PFS of metastatic NPC patients after first-line chemotherapy remains disappointing. The results of the present study suggest that for such patients, S1 MT can improve PFS and OS. Platinum-based chemotherapy is the preferred first-line treatment option for metastatic NPC (Chan et al. 2012; *NCCN Clinical Practice Guidelines in oncology (NCCN Guidelines). Head and Neck Cancers. Version 3.2021*, 2021), but the median PFS is only 2.7–7.2 months (Chan et al. 2005; Jin et al. 2012; Zhang et al. 2016). In the present study involving patients with disease control after first-line treatment, the non-MT group had a PFS of 9.3 months, which was longer than the 7.0 months with the GP regimen in a phase III clinical trial (Zhang et al. 2016). Nevertheless, the S1-MT group achieved a very significant PFS prolongation using S1 MT, reaching 16.9 months, and ultimately resulting in an OS benefit, consistent with the findings by Zhou et al. (2020) and Guo et al. (2020). In Zhou et al. (2020), the 2-year OS rate of patients receiving capecitabine/S1 MT reached 78.9%, significantly higher than the 62.7% observed in non-MT patients (*P* = 0.016). Even compared with the clinical study data of PD1 combined with GP followed by PD1 as first-line treatment (Mai et al. 2021; Yang et al. 2021), the S1-MT group in the present study still had better PFS. The median PFS was found to be 9.7 months in patients receiving camrelizumab combined with GP (Yang et al. 2021) and in patients receiving a toripalimab combination regimen (Mai et al. 2021). A longer MT can achieve more significant survival benefits through continuous tumour suppression. S1 MT for > 12 courses after standard treatment in N3 locally advanced NPC patients achieved better survival benefits (Zong et al. 2021). For metastatic NPC with capecitabine/S1 MT, the median PFS reached 27.6 months and MT lasting for 2 years or longer was recommended (Guo et al. 2020). The more significant OS benefit of the S1-MT group in the present study was present after > 2 years. This suggests that metastatic NPC patients can delay disease progression through longer MT and obtain better long-term survival.

Screening high-risk patients who need to continue suppression of their tumour burden will help to increase the effectiveness of MT. In a retrospective study, S-1/capecitabine maintenance therapy only prolonged the OS for locoregionally advanced NPC patients in the high-risk group (Zhu et al. 2021). The levels of and in changes of EBV-DNA and SAA in peripheral blood might be more sensitive than imaging examinations to changes in NPC burden than imaging examinations, suggesting the prognosis (Chan et al. 2017; Chen et al. 2020; Luo et al. 2016; Shen et al. 2015; Yip et al. 2014; You et al. 2019). Changes in the EBV-DNA load suggest that NPC progresses on average 2.3 months earlier than the imaging or pathology findings, and the sensitivity, specificity, and accuracy for distal metastasis were 91.1%, 80.0%, and 92.8%, respectively (Chen et al. 2020). In Zhou et al.’s study (2020), 48.2% of the patients with metastatic NPC tested positive for EBV-DNA after first-line standard chemotherapy, suggesting that half of those patients might potentially benefit from MT. Approximately 50% of patients in the present study still had positive EBV-DNA or continuously decreased SAA after first-line chemotherapy. Thus, NPC patients with detectable EBV-DNA after treatment will still be at high risk of disease progression and might benefit from subsequent treatments (Mai et al. 2021; Zhang et al. 2019; Zhou et al. 2020).

Because there is no consensus on the threshold for EBV-NDA positivity, the specific guidelines for EBV-DNA in MT are different. In a study conducted by Zhou et al. (2020), compared with patients who did not receive S1 MT, EBV-DNA-negative patients after first-line chemotherapy were more likely to benefit from MT in terms of OS (86.7% vs. 73%, *P* = 0.027), but the OS of EBV-DNA-positive patients did not improve significantly (49.5% vs. 55.4%, *P* = 0.824). However, in the present study, the patients all benefited from S1 MT to varying degrees regardless of their EBV-DNA status after first-line treatment, but EBV-DNA-positive patients benefited more significantly. In addition, the difficulty of homogenization of EBV-DNA detection (including reagent factors and technology-implementation factors) among different institutions is also the reason that affects the conclusions of various studies, including reagent factors, technology-implementation factors, etc. (Chan et al. 2018; Le et al. 2013). The EBV-DNA detection in this study was performed using reagents from Sun Yat-sen University, which has excellent research achievements in the field of NPC, and was conducted according to unified standards, which reduced the interference of technical factors with the results to a certain extent. More clinical data are needed to improve the clinical guiding role of EBV-NDA.

Although there are few data regarding SAA in metastatic NPC, studies monitoring NPC have shown that SAA tends to increase when NPC progresses, and the decrease in SAA is associated with treatment efficacy (Chen et al. 2018). A continuous decrease in SAA suggests that the tumour burden is present and potentially responds to chemotherapy. In the present study, although the adverse risks of PFS and OS were increased by nearly three times in patients with continuously decreased SAA, the PFS and OS were significantly improved due to continuous tumour suppression by S1 MT, suggesting that patients with continuously decreased SAA might be the population benefiting from S1 MT.

Good tolerability is an important factor for MT because of its long-term nature. As a derivative of 5-FU, S1 can cause adverse reactions that mainly include hyperpigmentation, mucositis, and hand and foot syndrome. The present study and other MT reports showed good tolerability (Zhou et al. 2020; Zong et al. 2019). The main adverse reactions of S1 MT (median MT of 24 courses) after concurrent chemoradiotherapy for locally advanced NPC were grade 1–2, and only 3/130 patients had a dose adjustment (Zong et al. 2019). In the present study, the median MT course was 23, and the maximum duration was 56 months; 13.6% of the patients received dose adjustment, consistent with the findings by Zong et al. (2019) and Guo et al. (2020). Reasonable dose adjustment is an important factor in enabling more patients to receive MT for a long time under the premise of ensuring the quality of life and might also be a key to achieving better outcomes.

However, the present study has several limitations. First, different first-line chemotherapy regimens might have a certain impact on the survival benefits of patients in the later stage. Although all the patients achieved disease control from the first-line chemotherapy, and the regimens were relatively uniform (the GP regimen accounted for more than 50% of cases). Second, quantified EBV-DNA and SAA might more accurately determine the population benefiting from MT. Therefore, further studies with larger sample sizes and stratified analyses will help develop better individualized MT in metastatic NPC patients. Finally, the choice of different 5-FU family drugs requires further study. Additionally, after immunotherapy has become the treatment option for metastatic NPC, PD-1 antibody as a choice strategy between maintenance treatment and oral S1/capecitabine maintenance regimen is also worthy of study.

In conclusion, S1 MT can significantly improve the PFS and OS of metastatic NPC patients with a response to first-line treatment and has good tolerability. After first-line treatment, EBV-DNA-positive patients and continuously decreased SAA might benefit more from MT than EBV-DNA-negative patients and those with stable SAA.

## Supplementary Information

Below is the link to the electronic supplementary material.

Supplementary file1 (DOCX 19 KB)

## Data Availability

The datasets generated during and/or analysed during the current study are available from the corresponding author on reasonable request.
