# Supplementary material for: Maintenance therapy improves the survival outcomes of patients with metastatic nasopharyngeal carcinoma responding to first-line chemotherapy: a multicentre, randomized controlled clinical study
Source: J Cancer Res Clin Oncol. 2022 Sep 8;149(8):4327–38. doi: 10.1007/s00432-022-04341-2 (PMC10349704; doi:10.1007/s00432-022-04341-2)
Supplement: Supplementary file 1 — Supplementary file1 (DOCX 19 KB) [file 432_2022_4341_MOESM1_ESM.docx]

**Table S1.** Univariable analysis of PFS and OS of patients with different EBV-DNA and SAA status in the two groups

|  | OS | | | PFS | | |
| --- | --- | --- | --- | --- | --- | --- |
|  | HR | 95% CI | P | HR | 95%CI | P |
| EBV DNA | | |  |  |  |  |
| S1-MT EBV+ | 0.393 | 0.227-0.681 | 0.001 | 0.600 | 0.373-0.965 | 0.035 |
| S1-MT EBV- | 0.570 | 0.338-0.962 | 0.035 | 0.740 | 0.461-1.189 | 0.214 |
| Non-MT EBV+ | 1.523 | 0.964-2.404 | 0.071 | 2.450 | 1.565-3.836 | <0.001 |
| Non-MT EBV- | Reference | |  |  |  |  |
| SAA |  |  |  |  |  |  |
| S1-MT SAA descend | 0.404 | 0.230-0.709 | 0.002 | 0.570 | 0.354-0.919 | 0.021 |
| S1-MT SAA stable | 0.695 | 0.430-1.125 | 0.139 | 0.726 | 0.465-1.131 | 0.157 |
| Non-MT SAA descend | 2.977 | 1.8784.721 | <0.001 | 2.866 | 1.834-4.478 | <0.001 |
| Non-MT SAA stable | Reference | |  |  |  |  |

OS: overall survival; PFS: progression-free survival; HR: hazard ratio; CI: confidence interval; MT: maintenance therapy; EBV: Epstein-Barr virus; SAA: serum amyloid A.

**Table S2.** Univariate analysis and multivariate analysis of the Cox risk ratio model for PFS- and OS-related outcomes in metastatic NPC patients benefiting from the first-line treatment

|  | Univariate analysis | | | Multivariate analysis | | |
| --- | --- | --- | --- | --- | --- | --- |
|  | HR | 95%CI | P | HR | 95%CI | P |
| **PFS** |  |  |  |  |  |  |
| Group (S1-MT vs Non-MT) | 0.045 | 0.327-0.634 | <0.001 | 0.326 | 0.255-0.473 | <0.001 |
| Age | 0.859 | 0.619-1.191 | 0.362 | 1.187 | 0.839-1.681 | 0.334 |
| Sex | 1.103 | 0.766-1.590 | 0.221 | 0.894 | 0.614-1.302 | 0.559 |
| Tumor metastasis site | 0.683 | 0.452-1.031 | 0.070 | 0.539 | 0.346-0.840 | 0.006 |
| Liver metastasis | 1.395 | 1.008-1.930 | 0.045 | 0.679 | 0.484-0.952 | 0.025 |
| ECOG of enrolling | 0.790 | 0.436-1.433 | 0.438 | 1.351 | 0.726-2.513 | 0.342 |
| First-line chemotherapy (GP vs. non-GP) | 0.869 | 0.695-1.086 | 0.336 | 0.884 | 0.713-1.096 | 0.261 |
| EBV-DNA status | 1.261 | 1.100-1.404 | 0.001 | 0.776 | 0.513-1.132 | 0.188 |
| SAA status | 1.257 | 1.097-1.440 | 0.001 | 0.729 | 0.495-1.073 | 0.109 |
| **OS** |  |  |  |  |  |  |
| Group (S1-MT vs Non-MT) | 0.485 | 0.320-0.735 | 0.001 | 0.375 | 0.239-0.590 | <0.001 |
| Age | 0.877 | 0.589-1.308 | 0.521 | 0.899 | 0.593-1.163 | 0.616 |
| Sex | 1.202 | 0.774-1.865 | 0.413 | 0.862 | 0.548-1.356 | 0.520 |
| Tumor metastasis site | 0.571 | 0.328-0.993 | 0.047 | 0.537 | 0.299-0.964 | 0.037 |
| Liver metastasis | 0.501 | 0.333-0.752 | 0.001 | 0.473 | 0.310-0.722 | 0.001 |

**Table S2.** Univariate analysis and multivariate analysis of the Cox risk ratio model for PFS- and OS-related outcomes in metastatic NPC patients benefiting from the first-line treatment (continued)

|  | Univariate analysis | | | Multivariate analysis | | |
| --- | --- | --- | --- | --- | --- | --- |
|  | HR | 95%CI | P | HR | 95%CI | P |
| ECOG of enrolling | 0.414 | 0.212-0.805 | 0.009 | 0.359 | 0.180-0.714 | 0.004 |
| First-line chemotherapy (GP vs. non-GP) | 1.049 | 0.854-1.314 | 0.714 | 1.070 | 0.834-1.372 | 0.596 |
| EBV-DNA status | 1.349 | 1.128-1.614 | 0.001 | 1.267 | 0.812-1.977 | 0.297 |
| SAA status | 1.200 | 1.012-1.422 | 0.036 | 0.621 | 0.392-0.984 | 0.042 |

PFS: progression-free survival; OS: overall survival; HR: hazard ratio; CI: confidence interval; ECOG: Eastern Cooperative Oncology Group; EBV: Epstein-Barr virus; SAA: serum amyloid A.
